# Spontaneous Coronary Artery Dissection/Intramural Haematoma in Young Women with ST-Elevation Myocardial Infarction: “It Is Not Always a Plaque Rupture Event”

**DOI:** 10.1155/2015/597234

**Published:** 2015-10-21

**Authors:** George Kassimis, Athanasios Manolis, Jonathan N. Townend

**Affiliations:** ^1^Department of Cardiology, Asklepeion General Hospital, Athens, Greece; ^2^Department of Cardiology, Queen Elizabeth Hospital, Birmingham, UK

## Abstract

Spontaneous coronary artery dissection (SCAD) is an unusual, but increasingly recognized, cause of ST-elevation myocardial infarction (STEMI), especially among younger patients without conventional risk factors for coronary artery disease (CAD). Although dissection of the coronary intima or media is a hallmark finding, hematoma formation within the vessel wall is often present. It remains unclear whether dissection or hematoma is the primary event, but both may cause luminal stenosis and occlusion. The diagnosis of SCAD is made principally with invasive coronary angiography, although adjunctive intracoronary imaging modalities may increase the diagnostic yield. In STEMI patients, the decision whether to pursue primary percutaneous coronary intervention (PCI) or appropriate conservative medical therapy is based on clinical presentation, the extent of the dissection, the critical anatomy involvement, and the amount of ischaemic myocardium at risk. In this case report, we present two cases of young women with SCAD and STEMI, successfully treated with primary PCI. We briefly illustrate the characteristic aspects of the angiographic presentation and intravascular ultrasound-guided treatment. SCAD should always be considered in young STEMI patients without conventional risk factors for CAD with primary angioplasty to be required in patients with ongoing myocardial ischemia.

## 1. Introduction

We present two cases of young women with spontaneous coronary artery dissection (SCAD) and ST-elevation myocardial infarction (STEMI) successfully treated with primary percutaneous coronary intervention (PCI).

## 2. Case  1

**Figure 1:**
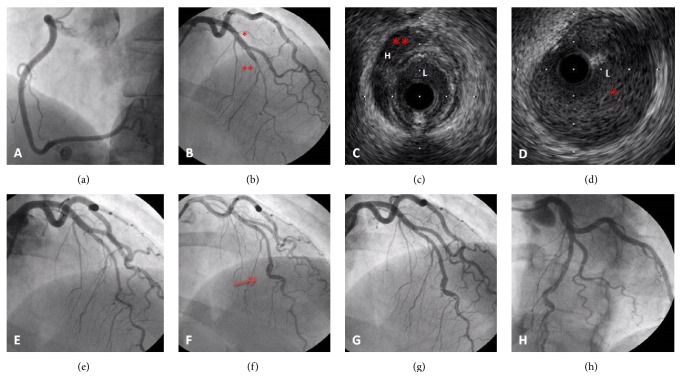
(a) Left anterior oblique (LAO) projection showing a favourable angiographic appearance of the RCA. (b) Right anterior oblique (RAO) cranial projection showing a long tubular stenosis of the mid-LAD, with abrupt demarcation (*∗∗*) from normal proximal segments (*∗*), which did not respond to intracoronary nitroglycerine. (c) IVUS examination showed near-circumferential hematoma (H) extending deep into the media and reducing the lumen (L). No atheroma was visualised. (d) IVUS examination of the proximal segments revealed normal vessel appearance and preserved lumen (L) caliber. (e) Final angiographic appearance of the mid-LAD, in RAO cranial projection. (f–h) At 6-month follow-up, RAO cranial projection of mid-LAD showing good coronary flow but with still evidence of significant vessel dissection in its distal course.

A 50-year-old postmenopausal woman with no cardiovascular risk factors was admitted with an anterior STEMI. The coronary angiogram (CA) demonstrated the right coronary artery (RCA) dominant and normal (Figure 1(a)); the left main stem and circumflex vessels all appeared normal, but there was a very unusual appearance in the mid left anterior descendent (LAD), of an almost subtotally occluded long tubular segment of LAD disease after a large diagonal branch (Figure 1(b)) with TIMI 2 coronary flow, which did not respond to 200 micrograms of intracoronary nitroglycerine.

There was a strong suspicion that this was an intramural haematoma (IH), rather than a plaque rupture event, and after predilatation with a 2/20 mm balloon at 8 atm, we performed intravascular ultrasound (IVUS) imaging. This clearly demonstrated that proximally and distally to the abnormal findings the vessel was entirely normal with no evidence of atheroma. However, there was a very long segment of about 70–80 mm in length of IH, which was compressing the true lumen (Figures 1(c) and 1(d)). After further predilatation with a 2.5/20 mm balloon at 10 atm and further 200 micrograms of intracoronary nitrate the flow picked up and the ST segments then settled and the patient became pain-free (Figure 1(e)). We electively did not stent the LAD due to the extensive length of the IH and the potential complications of stenting for IH including propagation of the haematoma both distally and proximally and because of the evidence from previous reports that, in many cases, spontaneous resolution and healing will occur with good luminal diameters. A postprocedural echocardiogram showed good left ventricular (LV) systolic function with no regional wall motion abnormality. The patient was discharged on dual antiplatelet therapy (DAPT) for twelve months and bisoprolol 2.5 mg o.d.

Six months later she was electively admitted for a follow-up CA to reevaluate the IH of LAD. The area of interest has been partially normalised; however there was still evidence of a significant dissection in its distal course (Figures 1(f)–1(h)). The patient was however asymptomatic and there was TIMI 3 flow in the LAD. We felt it would not be sensible to pass at this stage a coronary wire for intravascular imaging, as we feared that this might affect the LAD dissection and as such we stopped at this point. From a clinical point of view this lady remains very well without angina.

## 3. Case  2

**Figure 2:**
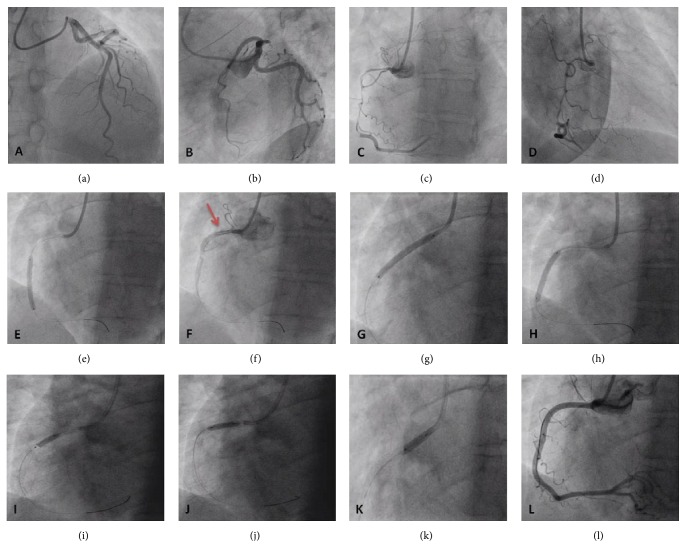
(a, b) RAO cranial and spider projections showing normal left coronary system. (c, d) LAO and RAO projections showing a very long tubular stenosis of the RCA, with abrupt demarcation from normal distal segments, which did not respond to intracoronary nitroglycerine and balloon predilation. (e) After the implantation of the first ZES 2.75/30 mm, there was a propagation of the dissection flap proximally (white line; red arrow (f)), successfully sealed with a second ZES 3/30 mm implanted proximally (g). (h) Postdilation with the stent balloon at the overlap of the 2 stents and with noncompliant balloon 3.25 mm proximally at 16 atm (i–k) with an excellent final angiographic result (l).

A 52-year-old postmenopausal woman with no cardiovascular risk factors was admitted with an inferior STEMI associated with temporary complete heart block. Urgent CA demonstrated an unobstructed left coronary system (Figures 2(a) and 2(b)) with an almost subtotally occluded long tubular abnormality within the RCA (Figures 2(c) and 2(d)), which did not respond to intracoronary nitroglycerine, with TIMI 2 flow. Primary PCI was successfully performed, with two zotarolimus eluting stents 2.75/30 mm and 3/30 mm implanted (distal to proximal) (Figures 2(e)–2(h)) and postdilated with a 3.25 mm noncompliant balloon at 16 atm (Figures 2(i)–2(k)) with an excellent angiographic result (Figure 2(l)). Intracoronary imaging was not performed, because the vessel was dissected till the ostium of the RCA, and there was a risk of exacerbating the disruption with the imaging catheter causing ostial vessel occlusion.

A postprocedural echocardiogram showed preserved LV systolic function. The patient was discharged on DAPT for twelve months and metoprolol 25 mg b.i.d. This lady remains completely asymptomatic 6 months after procedure.

## 4. Discussion

These cases highlight a condition that is a relatively rare cause of STEMI. Previous studies show that SCAD is commonest in the fifth decade, with a striking female predominance, particularly in the peripartum period, with 25–31% of reported cases occurring during this time. Other predisposing factors include connective-tissue and vasculitic disorders, cocaine abuse, and heavy isometric exercise and in some cases it has been described in association with oral contraceptive use in previously healthy individuals [1].

Urgent CA is indicated in STEMI patients and the definitive diagnosis of IH can be confirmed by IVUS or optical coherence tomography (OCT). However, this procedure is not always feasible, because of significant luminal compression by the extraluminal haematoma, and needs special care, as it can exacerbate the disruption of the vessel and cause vessel occlusion [2].

The two main pathological subsets have been described: with and without an intimal tear. An intimal tear may precipitate bleeding into the wall, with free communication between the true and false coronary lumens. Alternatively, a primary disruption of the “vasa vasorum” with subsequent bleeding and intramedial hemorrhage has been proposed as the underlying mechanism in patients in whom an intimal tear could not be identified. The resulting IH has no communication with the coronary lumen and causes luminal encroachment [1].

A simple angiographic classification to improve the diagnosis of SCAD was described by Saw [3]. Lesions with the hallmark of multiple lumen and/or contrast wall stain were classified as type 1 SCAD. Lesions with long, smooth tubular stenosis (representing IH as in our two cases) were classified as type 2 SCAD, which is seen in 2/3 of SCAD. Finally, lesions with focal or tubular stenosis (because of IH) that mimic atherosclerosis were classified as type 3 SCAD. The LAD is the most frequently involved vessel [1, 3].

The current gold-standard CA is excellent to assess luminal narrowing; however, it is poor in assessing the arterial wall, where the key abnormalities occur with SCAD. Intravascular imaging allows excellent visualizing of the arterial wall structure and composition. IVUS has a lower spatial resolution (150–200 *μ*m) but has deeper penetration allowing for the full vessel and extent of the IH to be visualized as in our first case. IVUS can delineate true and false lumens and detect IH, which appears as a homogenous collection behind the intimal-medial membrane. OCT, on the other hand, is a much higher resolution (10–20 *μ*m) modality and can visualize true lumen, false lumen, and even intimal tears and entry dissection points exceedingly well. However, it has poorer penetration than IVUS and may not visualize the full extent of the IH [4]. IVUS and OCT can also confirm guidewire position in the true lumen and assess optimal stent apposition and expansion [5].

Considerable controversy surrounds the aetiology of SCAD. Most likely this process occurs as a result of vascular shear stress and the presence of abnormal connective-tissue structure. Εosinophilic infiltrates observed in some cases may damage collagen and lead to cystic medial necrosis, and progesterone induced microstructural changes may be of importance in peripartum and contraceptive-associated SCAD [1]. Coronary artery tortuosity is highly prevalent in the SCAD population and is associated with recurrent SCAD [6, 7]. Angiographic features of SCAD are associated with extracoronary vasculopathy, including fibromuscular dysplasia (FMD), which could be a potentially causative factor [8, 9].

There is a lack of evidence supporting the value of specific pharmacological regimens or the value of systematic coronary revascularization over conservative management in SCAD-STEMI patients. Classically, thrombolytics and glycoprotein IIb/IIIa inhibitors are considered to be contraindicated, because they may promote additional intramural bleeding. The use of aspirin and clopidogrel following SCAD presentation is advocated by some but remains unsupported by clinical trials. The value of prasugrel or ticagrelor in these patients remains unknown. Beta-blockers are considered the mainstay of therapy, by reducing oxygen consumption and local shear stress, with the potential to stabilize the condition acutely and prevent late recurrence. Statins, while being a mainstay agent in typical atherosclerotic STEMI, are untested in SCAD [1].

Primary PCI should be considered in STEMI patients with proximal dissections in large vessels associated with ongoing ischemia and/or with reduced TIMI flow. However, PCI in patients with SCAD is associated with significant technical difficulties including propagation of intramural haematoma which may lead to further luminal compromise in remote segments including the left main stem. For this reason, it should perhaps be avoided if flow is good and there is no evidence of ongoing ischaemia or infarction. Residual distal coronary dissection and hematoma may sometimes be left untreated and will often heal with good late appearances [1, 9]. Whether drug-eluting stents (DES) provide any specific advantage over bare metal stents (BMS) in the management of SCAD remains unclear; however, as in our second case, DES may be preferred for long dissected segments in order to reduce the risk of restenosis [1]. Alternatively, BMS short spot stenting could be deployed with OCT guidance at the entry point of the IH as recently described [10].

Surgery should be considered in unstable patients with left main or 3-vessel involvement and also in patients with ischemia after a failed PCI [1]. However, a conservative strategy has also been proposed especially for selected patients with SCAD following initial clinical stabilization [11, 12]. Although long-term survival after an index SCAD episode appears better, compared to that for typical acute coronary syndrome, rates of major adverse cardiac events are similar [13].
